# Clarifying the paradigm for the ethics of donation and transplantation: Was 'dead' really so clear before organ donation?

**DOI:** 10.1186/1747-5341-2-18

**Published:** 2007-08-24

**Authors:** Sam D Shemie

**Affiliations:** 1 Division of Pediatric Critical Care, Extracorporeal Life Support Program, Montreal Children's Hospital, McGill University, Health Centre, Montreal, Quebec, Canada; 2 The Bertram Loeb Chair in Organ and Tissue Donation, Faculty of Arts, University of Ottawa, Ottawa, Ontario, Canada; 3 Donation Committee, Canadian Council for Donation and Transplantation, Edmonton, Alberta, Canada

## Abstract

Recent commentaries by Verheijde et al, Evans and Potts suggesting that donation after cardiac death practices routinely violate the dead donor rule are based on flawed presumptions. Cell biology, cardiopulmonary resuscitation, critical care life support technologies, donation and transplantation continue to inform concepts of life and death. The impact of oxygen deprivation to cells, organs and the brain is discussed in relation to death as a biological transition. In the face of advancing organ support and replacement technologies, the reversibility of cardiac arrest is now purely related to the context in which it occurs, in association to the availability and application of support systems to maintain oxygenated circulation. The 'complete and irreversible' lexicon commonly used in death discussions and legal statutes are ambiguous, indefinable and should be replaced by accurate terms. Criticism of controlled DCD on the basis of violating the dead donor rule, where autoresuscitation has not been described beyond 2 minutes, in which life support is withdrawn and CPR is not provided, is not valid. However, any post mortem intervention that re-establishes brain blood flow should be prohibited. In comparison to traditional practice, organ donation has forced the clarification of the diagnostic criteria for death and improved the rigour of the determinations.

## Commentary

Our ability to support organ failure with technology and transplantation raises important questions of when a disease is irreversible, when further treatment is no longer effective and when death has occurred. Continuing scientific advance forces our communities to reflect on the concept and definition of death, and we continue to thoughtfully struggle in this regard. The practice of organ donation galvanizes these issues. In particular, the immediacy of procurement in donation after cardiac death (DCD) has incited scrutiny and ominous concerns. Observations and criticisms of existing and evolving practices are indispensable, in order guard against erosions of ethical practice. This journal has contributed to the debate with recent provocative commentaries by Verheijde et al [1] followed by supportive responses by Evans [2] and Potts [3]. All three commentaries hinge much of their criticism around the dead donor rule, and the contention that current DCD practices violate this rule. This is the focus of the ensuing discussion.

Verheijde et al contend that this rule should be abandoned but transformed to allow the removal of organs from dying rather than dead persons. Evans contends that because complex organs taken from unequivocally dead people are not suitable for transplantation, human death has been redefined so that it can be certified at an earlier stage in the dying process. Potts categorically contends that DCD should be banned from practice. Central to this discussion is the distinction of whether the DCD candidate is recently and legitimately dead, as opposed to dying but nearly and not quite dead. The authors make allegations of transgressions of ethical and moral practice by the transplant communities.

Inherent to these contentions is the flawed presumption that there is and was a clear line between alive and dead and this was fully understood before and subsequent to organ donation practices. This line of 'unequivocal death', as implied by all 3 papers, was clearly delineated and is now clearly being violated. This erroneous presumption fuels much debate and misunderstanding about the complex biology of life and death. Death and our understanding of it as a biological event, with profound social, religious and psychological customs, is relative to the context of experience and the accumulation of scientific information available. This biological understanding has evolved and deepened as a direct result of technology, cell biology, organ donation and transplantation, but has been inadequately reconciled in law, health policy and bioethical discourse. Organ donation, as one of the immediate sequels to death, has forced the understanding, acceptance or persisting controversy of where that line is. For an instructive review, I refer readers to a superb historical, social and biological examination of death in a book entitled "The Way We Die" by Ivan and Melrose[4].

Historically, there has been little need for diagnostic or conceptual precision in regard to death. Early humans associated living with breathing and cessation of life was marked by unresponsiveness and the absence of respiration. The discovery of blood circulation by William Harvey in 1628 and the stethoscope in 1816 allowed the absence of heartbeat to be included in the determination. In recent decades and disturbingly so, the professional determination of death after cardiac arrest has remained rudimentary and of low rigour. Death occurred upon a doctor or coroner's determination. The criteria used were not articulated and remained untaught in training, ranging from absence of movement, breathing, heart sounds, pulse or EKG activity, applied at discretion of the attending physician. Observation and confirmation was not required and the irreversibility of death was not a practical concern, although diagnostic errors were made. Organ donation in general, and DCD in particular, has by necessity enhanced the rigour of the determination of death.

Life is fundamentally based on the maintenance of individual and collective cell function, dependent of the provision of nutrients and oxygen. Cell biology has demonstrated that a layer of human cells, separated from the human organism, may be grown in laboratory culture as long as they are bathed in a sterile supply of nutrients and oxygen. The human being, a complex arrangement of trillions of cells organized into organ systems, requires a cardiopulmonary delivery system (lung, heart and circulatory system) for oxygen and nutrients to reach the cells. The development and evolution of modern cardiopulmonary resuscitation evolving into cardiopulmonary support technologies have been important advances informing our concepts of life and death.

The most common mechanism of cell injury that leads to cell death is oxygen deprivation as seen with the arrest of blood flow. Oxygen deprivation causes the inability to produce energy and depletes energy stores required to maintain cell function. Within limits and differing according to which cell type is housed within which organ, the cell can compensate for energy loss and can return to normal function if the delivery of oxygen resumes. Permanent loss of oxygen delivery will cause cells to pass the threshold to irreversible injury and cell death, morphologically characterized by necrosis.

It is important to emphasize that the time to death for each cell, and each organ, will vastly differ. This has been well demonstrated by cell biologists who can remove and grow human cells in laboratory culture hours or days after death has occurred. Skins cells can be grown in culture when removed over 24 hours post-mortem [5], and brain cells can be grown if removed within 8 hours and can survive up to 78 days [6]. Transplant specialists, who are effectively organ biologists, have taught us that organ function is recoverable and transplantable for many hours post mortem, depending on duration of time after the arrest of circulation, temperature and the use of preservation solutions. Both in life and after death, different organs tolerate oxygen deprivation differently. The kidney is more resilient and resistant that other organs such as the liver. Within the brain, the cortex and cerebrum is less tolerant than the brainstem, resulting in conditions such as persistent vegetative states after resuscitated cardiac arrest. Even up to 7 days post-mortem, the human cornea can provide viable cells to enable transplantation [7]. From a purist perspective, the complete and irreversible cessation of all cell life has become increasingly indefinable.

Advances in organ support and replacement technologies teach us about the mechanics of death. Survival of the individual organs and the human organism is related to adequacy of oxygenated blood flow and this is the principle goal of cardiopulmonary resuscitation and critical care support. There are 3 basic mechanisms [8]: a) primary cardiac arrest leading to arrest of the circulation b) primary respiratory arrest, which via loss of oxygen causes a secondary cardiac arrest, or c) primary brain arrest, which via interruption of respiratory control causes a secondary respiratory then cardiac arrest. Regardless of initial disease state, all critical illnesses threaten life in this way. Interruption of this sequence and providing oxygen delivery with various forms of support is fundamental to critical care practice. Life sustaining technologies are deployed, with the use of artificial airways, mechanical ventilators, heart and circulatory support and kidney replacement therapies. Advanced support may include systems external to the body such as extracorporeal membrane oxygenation (ECMO) and artificial hearts (ventricular assist devices). The principle behind their application is to sustain vital function, to allow time or treatment to reverse the underlying life threatening state. When the underlying disease state cannot be remedied, the removal of those applied life sustaining technologies must occur for 'natural' death and cardiac arrest to ensue. Withdrawal and withholding of life sustaining technologies is the most common event preceding death in ICU practice worldwide.

The reversibility of cardiac arrest is now purely related to the context in which it occurs. The ability to restore the circulation depends on the location of the arrest, a predetermined ethical decision regarding level of medical intervention, the types of interventions available (cardiopulmonary resuscitation, extracorporeal membrane oxygenation, or ventricular assist devices), and the types of interventions actually used. Medicine has advanced to the point where all vital organs (heart, lung, liver, kidney) can be supported by machines, or replaced by transplantation. Complete and irreversible arrest of the heart is not death, as long as oxygenated circulation to the body can be provided mechanically. Circulation can be artificially maintained for days, weeks and months and the arrested heart can then be replaced by transplantation. The event may be the cardiac arrest, but death is only occurs if it leads to an accompanying loss of circulation.

The brain is the only organ that cannot be supported or replaced by technology. For all forms of severe brain injury, ICU care does not replace *any *functions of the brain. Breathing replacement machines merely interrupt the way brain failure leads to cardiac arrest. Contrary to previous perceptions that brain death invariably leads to cardiac arrest [9], any degree of brain failure, including brain death, can be sustained indefinitely with mechanical ventilation and vigilant care. While the brain may be irreversibly arrested, the body can be maintained, as demonstrated in case series of brain death in pregnancy with fetuses brought to term [10].

It is the arrest of brain blood flow that occurs after cardiac arrest that is of vital importance. The success of resuscitation is judged by the ability to reanimate the brain once spontaneous or mechanical circulation has been reestablished. The limits of brain resuscitation are commonly quoted as 4–10 minutes [4] and Verheijde et al [1] state the following, without reference or elaboration: 'longer than 10 minutes of absent circulation is required for irreversible cessation of the entire human brain, including brain stem function'. In reality, the duration of circulatory arrest that precludes recovery of any residual amount of brain function is unknown but is lengthening. Although arrest time is paramount, the conditions of the cardiac arrest (temperature) and the manner in which the circulation is re-established, (eg. hypertensive reperfusion, hypothermia, neuroprotective agents) will extend the time for potential recovery of various degrees of brain function well beyond 10 minutes [11]. This is supported by clinical studies of improvements in neurological function with the use of cooling in human cardiac arrest victims [12]. The aforementioned ability to grow human brain cells 8 hours after death suggests that at the cellular level, 'irreversible cessation of the entire brain' is elusive.

Brain death is better understood as brain arrest, characterized by the complete and irreversible loss of clinical brain function. The most reliable ancillary test for brain death is the absence of brain blood flow [13,14], related to pure oxygen deprivation. For the purposes of DCD, the cardiac arrest leads to absent brain blood flow. Although brain death examinations are not performed after circulatory arrest, the permanent absence of brain blood flow invariably leads to brain death in a short time frame and is conceptually and physiologically consistent with brain death [15]. In humans [16,17] and animal studies [18], it takes less than 20 seconds for cortical brain function to stop after cardiac arrest. This can be reversed if the brain blood flow is quickly re-established. It is not clear how long brain blood flow must be arrested to uniformly preclude reanimation of neurological function. However, any permanent absence of brain blood flow beyond 20 seconds will lead to permanent absence of brain function. Fundamentally, and most relevant to DCD, the issue is not whether the body or brain circulation and function can be resumed (because it can), but rather, whether it will be. The Institute of Medicine [19] and the ethics committee of the American College of Critical Care Medicine [20] have addressed the ambiguity surrounding the term "irreversible" similarly.

Accepting the concept that the permanent absence of brain blood flow is death, I share Verheijde et al concerns about the reported practices of CPR or extracorporeal oxygenated circulation applied after death, expressly for the purposes of organ preservation in DCD [22,22]. This is a true violation of the dead donor rule, as death cannot be ensured if brain blood flow recommences. For this reason, Canadian DCD guidelines explicitly prohibit any post mortem intervention that reestablishes brain blood flow [23].

A major criticism of DCD protocols has been the concern regarding the time of observation to determine death and the possibility that death is not 'irreversible' within the time limits proposed. There have been case reports of spontaneous resumption of heart function after cardiac arrest (autoresuscitation), ranging from seconds to minutes and longer. The true incidence and conditions that increase the potential for such an event are unclear and many reports are hampered by inadequate monitoring [24,25]. Some cases are related to mistaken diagnosis [26-28]. There is a clear distinction between those cases who have received CPR, where the reports of autoresuscitation range from seconds to 20 minutes [29]. No autoresuscitation after withdrawal of life sustaining treatment has been described beyond 2 minutes in the absence of CPR [30] suggesting that the provision of CPR is a confounding condition. This may occur because a buildup of pressure in the chest as a cause of absent circulation even as the heart is beating [24,31]. The incidence of autoresuscitation after even a minute, although cited as a common concern and criticism, is extremely rare and is likely negligible. Regardless, there have been no prospective studies to substantiate or negate these concerns. The true incidence, risk factors, temporal characteristics and outcomes after autoresuscitation are unknown. It has been estimated that a study of over 10,000 patients would be required to have sufficient power to exclude the possibility of autoresuscitation after more than 2 minutes. At minimum, the current discussion and criticisms of DCD as manifest in the preceding commentaries [1-3] should distinguish between controlled and uncontrolled DCD. The vast majority of DCD worldwide is controlled, in situations where life support is withdrawn and CPR is not provided. Criticism of controlled DCD on the basis of violating the dead donor rule, where autoresuscitation has not been described beyond 2 minutes, is not valid.

From a cellular, organ and whole body human-based perspectives, the commonly used terms in the lexicon of death, such as 'complete and irreversible' cessation [32,33] of function or life processes are not definable. The so-called time of death has always been an arbitrary moment within an overlapping segment of decreasing vital functions and increasing quantity of cell death [4]. No matter how convenient it is to assume that death and life are opposite and that a patient is either dead or alive, the process of death is a gradual event where organs and cells die at different rates depending on their resistance to the lack of oxygen [4]. As a result, the biology of death cannot be a moment, as the law may imply and people wish to believe. It is and always has been a line within an overlapping segment of decreasing cell functions and increasing cell death, based on the existing methods for its determination and now further complicated by available methods of circulatory support.

In medical practice and law, the separation between being alive and dead should not be ambiguous. It marks the point in time after which consequences occur, including no legal or medical requirement to provide resuscitation or life support technologies, loss of personhood and most individual rights, the opportunity for organ donation and autopsy proceedings, execution of the decedent's legal will, estate and property transfer, payment of life insurance, final disposition of the body by burial or cremation, and religious or social ceremonies to mark the end of a life. Organ donation has not created this reality, but continues to force its reconciliation.
